# A Lung Cancer Patient’s Passionate Desire for Sexual Healing

**DOI:** 10.1093/oncolo/oyad268

**Published:** 2023-10-24

**Authors:** Annabelle Gurwitch

## Abstract

Most female patients with lung cancer experience sexual dysfunction. This narrative describes one patient’s experience, focusing on how tobetter address issues related to toxicities stemming from long term treatment on targeted therapies.

Diagnosed in 2020 at stage IV, I was single, with an incurable cancer, and along with the likelihood of a shortened life span, I mourned the loss of intimacy this portended. Lung cancer inevitably evades treatment, but *if* I tested positive for a genetic mutation for which a corresponding biomarker therapy was available, *if* I could tolerate the medication, and *if* my cancer responded, progression could be temporarily stalled. I got lucky on the trifecta of precarious conditions that provide a temporary reprieve. When I hit one year of stability, I began to hope to eke out a few more purposeful, and dare I dream, pleasurable years?

After reconnecting with an old crush undeterred by my prognosis, I came down with a UTI. At the appointment with my gynecologist, I was doubled over with discomfort, but smiling, nonetheless. “You have honeymoon cystitis,” was how he put it. I am deeply fond of this doctor, whom I have seen for 2 decades, but it is baffling that this term, informed by Victorian era notions of chastity, assuming that the first time a woman would have sexual intercourse would be on her honeymoon, is regularly invoked by medical professionals.

“Cancer is my bad boyfriend and I’m cheating on him with joy!” I giggled like a teenager.

“Just give it a rest for a few days.”

I threw back a round of antibiotics, stockpiled cranberry juice and a trifecta of pro-, pre-, and post-biotics. But when I came down with another UTI, and then another, I asked my cancer care team if there might be a connection between my targeted therapy and the infections. They maintained that they were unaware of any correlation. After 6 months of averaging 2 UTIs a month, the relationship had blossomed, but I began to worry that my partner and I might have some sort of bacterial incompatibility that recommended breaking it off. Again, I asked my oncologist about a possible connection. “Stop having so much sex” I was advised with a dismissive shrug. The implication was as demoralizing as it was clear—just be happy you are still alive.

Then, in August 2022, after giving a patient advocate address at the International Association for the Study of Lung Cancer conference in Vienna, I attended a presentation recommended by Dr. Brandon Stiles, a thoracic surgeon at Montefiore, Albert Einstein College of Medicine. Dr. Narjust Florez, Associate Director of The Cancer Care Equity Program and thoracic oncologist at Dana- Farber Brigham Cancer Center presented data from the Sexual Health Assessment in Women with Lung Cancer, SHAWL Study, for which she was the lead author. Scheduled at 7:30 AM, a decidedly unsexy time, I was the person punctuating her talk with fist bumps, weeping, and barking, “Yes!” as I learned that 77% of female lung cancer patients experience moderate to severe sexual dysfunction. When the call came for questions and comments, there were no takers. I stood up, as though at a 12-step meeting, and introduced myself. “I’m Annabelle Gurwitch, and I have a very, very dry vagina. Thank you for bringing attention to this issue, I thought I was alone.”

As to why our symptoms go unaddressed, the study points to numerous reasons, including a cultural taboo surrounding discussions of sexual health. Florez, herself, was unaware that female patients with lung cancer were experiencing UTIs and painful intercourse (it was not brought up in training), until in 2019, she noticed a patient’s armpit rash. The patient, in her early 30s, and her partner had been having intercourse in her armpit because vaginal penetration caused extreme discomfit. The patient had been embarrassed to bring the topic up, in addition to conditioning to the well-documented history of female patients’ complaints falling on deaf ears. Can you imagine a man in treatment for prostate cancer being told, like I was, to just stop having sex? You cannot, because it does not happen; monitoring sexual health is standard protocol in prostate cancer management.

“We’re trained to pay attention to the effects of therapies on patients’ other organs,” Dr. Florez told me, “And, it’s tragic because a vagina is not only an important organ, but provides our patients with pleasure. You can’t say that about a spleen.”

Female sexual health, understandably, presents a relatively new set of issues for both patients and long-time practitioners in lung cancer as City of Hope thoracic oncologist Jack West recently reminded me. Prior to 2017, when targeted therapies became standard first line treatment, the 5-year survival rate was 4%-7%, compared to 23%-40% which it hovers around presently. As the only treatments carried high toxicities, “sexual activity wasn’t top of mind” for most of his patients, and if side effects didn’t send someone to the ER at 1 AM, he was not focused on them either. In this new era, he noted, “Chronic, non-emergent side effects should be the focus of more of our attention—as we have treatments that are effective enough for people to be living much longer, they really affect quality of life.”

Also, in the past, patients were overwhelmingly male. For the first time in history, more younger women in the US will get diagnosed than young males. In patients less than 50 years old, 2/3 are young women. Preserving fertility and sexual dysfunction are top of mind for this population but the SHAWL study finds that for almost 80% of us, our providers have not yet registered this new reality.

As Florez explained to me, 2 of my side effects (considered low-level toxicities) were conspiring with my canoodling to produce the UTIs. Introducing friction causes small tears in the delicate lining of the vagina, which was already compromised due to the tyrosine kinase inhibitors blocking collagen production. Add in *E. coli* bacteria, a by-product of chronic diarrhea, another TKI side effect, and you have got the perfect climate for persistent UTIs. Granted, it is possible that a contributing factor to my doctor’s not recognizing the relationship between my side effects was due to widespread underreporting by patients. A common topic of discussion within the patient community is the fear of being taken off of our medications.

I can testify to my own reluctance to disclose the severity of side effects at the onset of treatment. At the 3-month mark, the first assessment of the targeted therapy I was receiving, CT scans of my lungs showed shrinkage of the main tumors and the trace areas were diminished as well. The oncologist was pleased. I was miserable. Fatigue was possible, I’d been warned, but nothing prepared me for the level of enervation I was experiencing. If I got up in the morning and made coffee, the exertion of operating the machine sent me back to bed. If I sat down at my desk, by the time I powered up my computer, I’d need another nap. There were days when I had a good 6 or 7 hours of wakefulness, unfortunately, none of those hours were consecutive.

One day, I summoned the strength for an afternoon walk. I was less than 3 blocks from home when I could not take another step. I crumpled like a wet potato chip onto a grassy meridian. I lay there, inert, and unable to rouse myself, for maybe 30 minutes, until I managed a form of locomotion, something akin to an injured racoon. Not a single car that passed stopped to inquire if I was OK. “Women in Los Angeles will do anything to stay fit, I saw a woman crawling on all fours on the sidewalk today!”

Admitting to the severity of the fatigue might risk burning off my first-line treatment; on the other hand, I was not sure that the way I was surviving qualified as living. After confiding in my doctor, each day of a 3-month trial period at a lower dosage was a nail biter. Thankfully, the stability held.

With a new care team and through mitigations suggested by Florez, I have halved my frequency of UTIs, lessening my risk of becoming antibiotic resistant, while still getting my sexy on.

Intimacy, of course, can be fostered in many forms. Jill Feldman, co-founder of EGFR Resisters, a lung cancer advocacy group, and a co-sponsor of the SHAWL study, reminded me, “Emotional intimacy is important too! Initially, after I was diagnosed, my husband and I were focused on the well-being of our four kids. We also share a love of the Kansas Jayhawks, so we started doing date nights to the Final Four. It was so liberating and brought us back to us, fun, adventure, and far away from cancer.” Me? I am with Marvin Gaye. I’d like some of that “sexual healing.”

It’s widely recognized in the oncology community that it was breast cancer survivors who pushed for improvements in survivorship issues like insurance coverage of breast reconstruction and conversely the normalization of the “going flat” movement by medical practitioners who were initially reluctant to endorse this choice. Our pink warrior sisters are providing leadership in another area as well: rethinking methodologies used in toxicity level reporting during clinical trials. In 2021, the Patient Centered Dosing Initiative, founded by breast cancer survivor Anne Loeser, conducted surveys confirming that disparities in language used in patient surveys and clinician reports during clinical trial data collection contributed to “oncologists somewhat underestimate(ing) the prevalence and severity of their patients’ side effects.” Since the results were presented at ASCO in 2021, the FDA has launched an initiative, Project Optimus, that holds promise for improving patient-provider communication.

I am coming up on 3 years in treatment and any indifference I have experienced in Cancerland has been off-set by the calamity of kindness I have received from members of the medical establishment. Dr. Florez is an extraordinary evangelizer for our vaginal health and has earned a Swifty-like following in the female lung cancer patient community, but I am resolutely hopeful that once we gain wider acknowledgement of the issue, our care teams will respond, as well, and introduce sexual health into regular consultations. Modalities like the Ex-Plissit Model, which offers guidance for the introduction of topics related to sexual health, are currently in use in medical settings. However, this training was not included in any curriculum of the oncologists I spoke with.

In her presentations, Florez often gesticulates with an intimate accessory or tube of favorite lubricant, props she regularly offers to her audiences with assurances that though we live in a share economy, these are virginal—so to speak. “So, are there always takers?” I asked Florez. “Always.” “And, what’s the ratio of patients and clinicians?” “One hundred percent patients,” she answered definitively, no need to consult her notes on that data point. Perhaps the day that clinicians leave her presentations toting unmarked brown paper bags will signal a small bit of progress.

## Notes

Annabelle Gurwitch is a 2-time finalist for the Thurber Prize for American Humor Writing and a New York Times Bestselling author of 5 books, including “You’re Leaving When? Adventures in Downward Mobility,” a New York Times Favorite Book for Healthy Living. Her writing appears in the New York Times, Washington Post, and Los Angeles Times. She is a member of the International Association of the Study of Lung Cancer and sits on the Editorial Board of Cancer.Net, the patient information website of the American Society of Clinical Oncology (ASCO).

